# Behavior Change Intervention for Smokeless Tobacco Cessation Delivered Through Dentists in Dental Settings: A Pragmatic Pilot Trial

**DOI:** 10.1093/ntr/ntad243

**Published:** 2023-12-11

**Authors:** Shaista Rasool, Richard Holliday, Zohaib Khan, Fiona Dobbie, Linda Bauld

**Affiliations:** Usher Institute, The University of Edinburgh, Scotland, UK; Insitute of Public Health, Khyber Medical University, Peshawar, Pakistan; School of Dental Sciences, Faculty of Medical Sciences, Newcastle University, Newcastle upon Tyne, UK; Insitute of Public Health, Khyber Medical University, Peshawar, Pakistan; Usher Institute, The University of Edinburgh, Scotland, UK; Usher Institute, The University of Edinburgh, Scotland, UK

## Abstract

**Introduction:**

Evidence on smokeless tobacco (ST) cessation interventions is scarce. The South Asian (SA) region that shares more than 90% of the burden of ST use is grossly underrepresented in research on ST cessation. This study aimed to assess the feasibility of delivering and investigating a behavioral support intervention for ST cessation in dental settings in Pakistan.

**Methods:**

A multicenter, pilot, two-armed parallel-group, individually randomized control trial, with a 1:1 allocation ratio, was conducted at two dental hospitals. Eligibility criteria included being an ST user seeking dental treatment and not currently accessing cessation support. All participants were provided written self-help ST cessation material. The intervention group also received a dentist-delivered, bespoke behavioral support intervention for ST cessation developed for users of SA origin. Participants were followed up telephonically at 3 and 6 months. Self-reported 6-month abstinence was verified by salivary cotinine. Analysis was descriptive, with 95% confidence intervals presented where appropriate.

**Results:**

One hundred participants were successfully recruited from the selected hospitals. Of these, 78% continued to engage throughout the study duration and provided primary outcome data, whereas 63% completed all hospital visits. The outcome measures were successfully collected. Biochemically verified 6-month abstinence in the intervention and control groups was 10% and 4%.

**Conclusions:**

It was feasible to deliver and evaluate a dentist-delivered behavioral support intervention for ST cessation in Pakistan. The data suggested that the intervention may improve ST quit rates. The findings of this study will be useful in informing the design of future definitive studies.

**Implications:**

To our knowledge, this is the first pragmatic pilot trial on ST cessation in dental settings in Pakistan and the first trial on dentist-delivered structured behavioral support intervention for ST cessation. It adds to the scarce, trial evidence based on ST cessation interventions. The findings suggest behavioral support intervention for ST cessation may improve quit rates. The trial was conducted in a country with poor ST control measures, where ST products are not taxed, the products are sold openly to and by minors, and the users are offered negligible cessation support. The findings may, therefore, be generalizable to low–middle-income countries, particularly SA countries, with similar policy backgrounds.

## Introduction

Despite substantial global progress in the implementation of tobacco control measures guided by the “Framework Convention on Tobacco Control” (FCTC), smokeless tobacco (ST) control considerably lags behind in comparison to the control of combustible tobacco.^1^ The use of ST is reported in 127 countries; however, the bulk of the burden is largely shared by the South Asian (SA) region.^2–4^ Widely promoted as a less harmful substitute for cigarette smoking and essentially considered as a problem exclusive to the SA region, ST has received very limited attention from policy makers even in regions sharing the greatest burden.^1^

Despite being a signatory of the FCTC since 2005, Pakistan (an SA country with a high use of ST) lags behind its neighboring countries in terms of ST control laws and alignment of the existing laws with FCTC guidelines. According to the latest Global Adult Survey (GATS), the overall prevalence of tobacco use (smoked and smokeless) in Pakistan is 19.1% (12.4% smoked and 7.7% ST).^5^ The latest national figures indicate a rise in the use of ST (9%) (15% males and 3% females).^6^ ST use is deeply embedded in the social and cultural fabric of South Asia, where its use is widely associated with socializing, sharing, and family tradition.^7,8^ In addition to these social influences, there are individual-level factors that influence ST use, such as sociodemographic factors like age, gender, education, and income, and a wide disparity in the gender of tobacco users exists in Pakistan. However, the disparity is lower for ST use as compared to the use of smoked tobacco (male vs. female use of ST is 11.4% vs. 3.7% and male vs. female use of smoked tobacco is 22.2% vs. 2.1%).^5^The prevalence of ST in Pakistan is inversely related to education and income, with a higher use among individuals with no formal education and lower income.^5^

As with most countries, ST control in Pakistan remains largely neglected against the backdrop of the challenges that Pakistan faces with the implementation and enforcement of key measures for smoked tobacco.^9^ Cessation services was one of the four key policy instruments identified in a recent paper that explored the policy challenges, opportunities, and priorities for ST control in Pakistan.^9^ While Pakistan, like other low–middle-income countries (LMICs), struggles to prioritize its funding and resources, it might not be possible to shift priorities.^10^ However, the existing health system (such as dental settings) can be effectively engaged to extend its role to tobacco control with minimum investment. Every year more than 60% of tobacco users visit their oral health care providers (OHPs), which places them in a unique position to effectively contribute toward reducing the prevalence of tobacco use, whether that is by directing tobacco users to cessation services, or by engaging with patients in cessation counseling.^11^ While there is no reliable data on the utilization of or access to oral health care in Pakistan, there is evidence of the dearth of dental health services in Pakistan. With more than 90% of oral health problems unresolved, there are overall 1.2 dentists per 100 000 population and 1 dentist per 200 000 in the rural areas.^12^ The scarcity of services, in turn, leads to greater patient flow at the available dental care facilities, especially the public sector facilities which charge patients at a nominal rate. Furthermore, while oral health care is declared to be part of the primary health care system, it is the tertiary-level hospitals that serve as the primary nidus of dental care for the masses in Pakistan, where primary dental care is only marginally covered by the weak health system. These hospitals function as primary, secondary, and tertiary care for dental patients and present themselves as an ideal candidate for tobacco cessation services as far as reach to patients is concerned.

All OHPs are recommended to provide support for tobacco cessation during routine dental care (WHO FCTC article 14).^13,14^ The OHPs are among the first to notice any changes that can occur in the oral cavity, due to tobacco use. While there is evidence on the effectiveness of tobacco cessation interventions, delivered by OHPs in achieving long-term abstinence, the bulk of this evidence, is based on research from high-income counties.^15^ For instance, the latest Cochrane review on tobacco cessation interventions via dental professionals, included six randomized control trials on ST cessation and none of these was conducted in the SA region (all six were conducted in the United States).^15^ This clearly limits the generalizability of the findings and highlights the need for more evidence from LMICs, which share the greatest burden of the problem.

While this lack of evidence on ST cessation interventions is more pronounced in dental settings or via OHPs, there is overall limited evidence from the SA region on cessation interventions. It is also important to mention that while there is a growing recognition of the need for ST cessation support in the SA region and a growing body of evidence from the region,^16–21^ not a single study included in the latest Cochrane review investigating interventions for ST cessation was from this region (or from any LMIC).^22^ While the authors reported evidence of benefit in favor of behavioral support and pharmacotherapy for ST cessation, a need for further research, especially from regions not previously represented, was highlighted. This multicenter feasibility study, therefore, aimed to assess the feasibility of delivering and investigating a dentist-delivered behavioral support intervention for ST cessation in Pakistan, a low–middle-income SA country with a high burden of ST use.^5,23–25^ The aim was to assess the eligibility, recruitment and retention rates, and the feasibility of collection of data on ST outcome measures, using a randomized controlled design.^26^

## Materials and Methods

The detailed methods of the study are published in a protocol paper elsewhere.^26^ Briefly, this was a multicenter, individually randomized, two-armed parallel-group, pilot trial with a 1:1 allocation ratio, conducted at two dental hospitals in Khyber Pakhtunkhwa (KP) (the northwestern province of Pakistan). Participants were dental patients who were ST users (18 years and above); seeking dental care at the selected hospitals (and selected departments), not accessing cessation support and willing, and able to provide written or thumbprint informed consent (Appendix 1). Both randomization groups received written, self-help material on ST cessation and the intervention group received a structured behavioral support intervention for ST cessation from dentists, which was delivered in three face-to-face sessions.

A favorable ethics opinion was obtained from the Edinburgh Medical School, Research Ethics Committee (REC; 06/09/2021; 21-EMREC-024), and from local bodies in Pakistan, including REC of Khyber Medical University (KMU) and REC of Khyber College of Dentistry (KCD), Pakistan. This trial was registered prospectively (ISRCTN1807210).^26^ The study adhered to the CONSORT guidance for pilot and feasibility trials^27,28^ and a completed CONSORT checklist is included as Appendix 2. Protocol amendments are included in Appendix 3.

### Setting

The study was conducted at three specialty departments (periodontics, prosthodontics, and endodontics) of two tertiary care hospitals in KP. The selected hospitals included a public and private sector, tertiary care, and dental teaching hospital: Khyber College of Dentistry (KCD), public, and Sardar Begum Dental College (SBDC), private. The choice of recruitment of dental patients from periodontics and prosthodontics was based on practical as well as clinical grounds, following preliminary discussions with dentists working at the study sites.^29,30^ The Department of Endodontics was included in the trial a week after commencement of recruitment to accelerate the recruitment rate. (Start of recruitment coincided with the third COVID-19 peak in Pakistan, due to which recruitment in the first week was slower than expected.)

### Identification and Recruitment

Patients: Potential participants were identified during initial oral examination, by the dentists working at the selected departments. Once identified, the research team was notified, which then discussed the study and invited them into the trial, before seeking written informed consent.

Participating dentists: All permanent faculty members and trainee medical officers, working in the departments of prosthodontics and periodontics, were invited, face-to-face, by the first author (SR) for participation in the trial (for intervention delivery).

A copy of the signed informed consent form was given to all trial participants for their records.

### Sample Size

In line with the recommendations for pilot trials, no formal sample size calculation was completed and we planned for 30 participants in each study arm.^31,32^ Anticipating an attrition rate of 36% (from a previous trial testing the same intervention),^16^ we, therefore, planned to recruit 100 participants (50 in each arm of the trial).

### Interventions

Participants in the intervention group received a structured behavioral support intervention for ST cessation, developed for users of the SA origin “behaviour support for smokeless tobacco users of SA origin” (BISCA).^16^ BISCA was delivered by dentists in three sessions, namely prequit, quit, and postquit. All sessions involved face-to-face counseling with the aid of a flipbook, which contained interactive messages for the participants to view on one side and prompts on the other side, for the dentists to guide the conversation with the patients. A TIDierR checklist for the intervention is provided in Appendix 4.

The participants in the control group were given written self-help material by the dentist, in the form of a booklet containing tobacco cessation messages. A TIDierR checklist of the self-help material is provided in Appendix 5.

All participating dentists attended a 1-day training workshop on intervention delivery.

### Assignment of Interventions

We randomly allocated the participants to the control or intervention group, after the assessment of eligibility and completion of informed consent. Table 1 provides an overview of the data collection flow and trial visits for the two groups. The randomization was done in a 1:1 ratio, using random permuted blocks of variable length. To achieve assignment concealment, an independent statistician at the clinical trial unit of KMU. who had no involvement in the study, generated the allocation schedule. The allocation schedule was contained in sealed opaque envelopes each bearing on the outside a unique number for each participant. The sealed envelopes were accessible only to SR, opening them, only after obtaining written informed consent.

**Table 1.** Data Collection Flow and Overview of Trial Visits and Follow-ups

Visits/follow-up	Control	Intervention
Baseline (visit1)	Consent Demographics Baseline measurements (self-reported tobacco use and dependence scales) Randomization Routine dental treatment Self-help material	Consent Demographics Baseline measurements (self-reported tobacco use and dependence scales) Randomization Routine dental care ST cessation intervention (prequit session with dentist involving face-to-face counseling and take home self-help material)
Visit 2	ST-related outcome measures (self-reported ST use) Routine dental treatment	ST-related outcome measures (self-reported ST use) Routine dental care ST cessation intervention (quit session with dentist involving face-to-face counseling and take-home calendar for monitoring ST use)
Visit 3	ST-related outcome measures (self-reported tobacco use and dependence scales) Routine dental treatment	ST-related outcome measures (self-reported use and dependence scales) Routine dental care ST cessation intervention (postquit session with dentist involving face-to-face counseling and return of calendars)
3-mo follow-up (telephone)	ST-related outcome measures (self-reported use)	ST-related outcome measures (self-reported use)
6-mo follow-up (telephone)	ST-related outcome measures (self-reported use)	ST-related outcome measures (self-reported use)
Visit 4	Saliva sample collection (only for those participants who self-reported 6-mo abstinence)	Saliva sample collection (only for those participants who self-reported 6-mo abstinence)

SD = standard deviation; ST = smokeless tobacco

### Concomitant Care

All participants continued to have their dental treatment as usual.

### Blinding

Blinding was not possible due to the nature of the intervention provided.

### Outcomes and Data Collection Methods

The outcome measures used to assess feasibility included eligibility, recruitment and retention rates, compliance with the intervention, and collection of data on ST use being rehearsed for future definitive trials. The outcome measures used to assess ST use included self-reported ST use, nicotine dependence measures “Fagerstörm Tobacco and Nicotine Dependency Scale for Smokeless Tobacco” (FTND-ST) and “Oklahoma Scale for Smokeless Tobacco Dependence” (OSSTD)^33,34^, and salivary cotinine (SC).^35,36^

### Follow-Up

We followed up the participants telephonically at 3 and 6 months from the third hospital visit. For the participants who failed to attend the third visit, their second visit was used as the reference with 2 weeks added for the 3-month follow-up. Whereas, if they failed to attend both the second and third visit, then their first visit was used as the reference with 1 month added.

Following up the participants for 6 months was based upon the Russell Standards for smoking cessation studies.^36^ Those who could not be followed up at 6 months were deemed lost to follow-up and considered as continuing ST use or to have relapsed, in line with standard research practice^36,37^

### Data Analysis

Data analysis was descriptive and in line with the recommendation for feasibility and pilot trials.^32,38^ Proportions/rates were reported with 95% confidence intervals (CIs). Quantitative outcome measures were reported as means and standard deviations (SDs) with 95% CIs. Missing data were not imputed for frequency and quantity of use. Data were analyzed in STATA 14.

## Results

**Figure 1.**
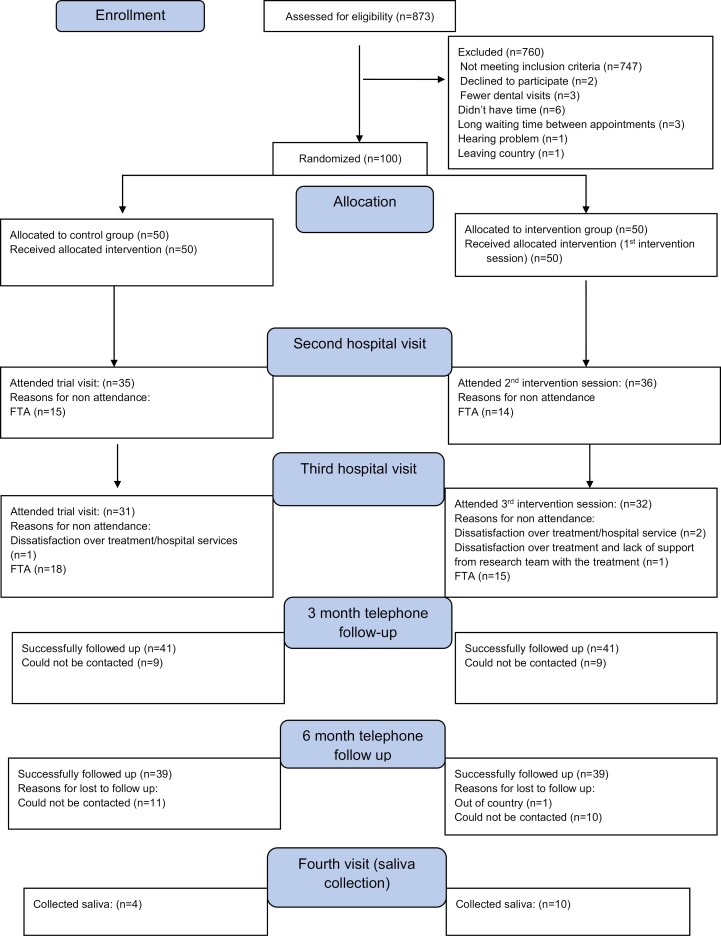
CONSORT flow diagram of the trial.

One hundred and thirteen potentially eligible participants were identified over the 5-week (+2 day) recruitment period (25/01/2022 to 02/03/2022). Of these, 100 were found to be eligible and enrolled in the study (60 from KCD and 40 from SBDC). The eligibility rates at KCD and SBDC were 14.5% (95% CI: 10.0, 18.9) and 11.5% (95% CI: 5.1, 17.8), respectively. Recruitment at SBDC started a week earlier than KCD and lasted a week longer. The overall consent rate was 88.4% (95% CI: 89.5%, 86.9%). A slight difference was observed between consent rate at KCD and SBDC, with a mean consent rate at KCD of 89.4% (95% CI: 83.2, 95.7) and at SBDC of 81.3% (95% CI: 63.7, 99.0). Data collection for the trial was completed on December 16, 2022, when the last saliva sample was collected. Figure 1 shows the CONSORT flow diagram for the study.

Twenty-one dentists agreed to participate from the 31 dentists invited. Five dentists dropped out from the study; of these, four dropped out due to a change in their duty to another department/hospital, and these four dentists attended the intervention training workshop. Whereas, one dentist dropped out before the training workshop, without stating any reason. The remaining 16 dentists participated in the trial. Amongst these 16, 2 dentists (who were the senior most) did not deliver the intervention.

### Participant Baseline Characteristics

The trial sample (*n* = 100) consisted of 99 (99%) males and 1 female participant (1%). Ethnicity was predominantly Pashtun (Pashtu-speaking natives belonging to KP) (*n* = 99, 99%), one participant was “Chitrali” belonging to district Chitral of KP. A summary of demographics by randomization is described in Table 2. The demographic profile of the sample was a close representation of the ST users belonging to this region; however, only one female was recruited. Since the female use of ST in Pakistan is 3.7%, the female representation in the sample should have been higher.

**Table 2.** Participant Characteristics at Baseline

	Control	Intervention	Total
Sex (*n*/%)			
Female	0 (0%)	1 (2%)	1 (1%)
Male	50 (100%)	49 (98%)	99 (99%)
Education (*n*/%)			
No education	19 (38.7%)	16 (33.3%)	35 (36.0%)
Grades1–4	1 (2.0%)	2 (4.1%)	3 (3.0%)
Primary	7 (14.2%)	1 (2.0%)	8 (8.2%)
Middle	3 (6.1%)	10 (20.8%)	13 (13.4%)
Secondary	8 (16.3%)	4 (8.3%)	12 (12.3%)
Higher Secondary	3 (6.1%)	6 (12.5%)	9 (9.2%)
Bachelors	6 (12.2%)	8 (16.6%)	14 (14.4%)
Masters	2 (4.0%)	1 (2.0%)	3 (3.0%)
Occupation (*n*/%)			
Unemployed	2 (4.2%)	1(2.0%)	3 (3.1%)
Retired	2(4.2%)	2 (4.1%)	4 (4.2%)
Full-time student	5(10.6%)	6 (12.5%)	11 (11.5%)
Managerial/professional occupation	-	4 (8.3%)	4 (4.2%)
Routine/manual occupation	30 (63.8%)	25 (52.0%)	55 (57.8%)
Intermediate occupation	4 (8.51%)	7 (14.5%)	11 (12.6%)
Self-employed	4 (8.51%)	3 (6.2%)	7 (7.3%)
Type of tobacco used (n/%)			
Smokeless tobacco	41(82%)	39 (78%)	80 (80%)
Smokeless and smoked tobacco	9 (18%)	11 (22%)	20 (20%)
Smokeless tobacco product used (*n*/%)	50 (100%)	50 (100%)	100 (100%)
Naswar Other			
Pattern of using ST (*n*/%)			
Daily	49 (98%)	47 (94%)	96 (96%)
Someday	1 (2%)	3 (6%)	4 (4%)
Age (years) mean (SD)	42.4(16.7)	40.2 (17.4)	41.3(17.0)
Self-reported daily frequency of ST use (number of dips/day)	15.3(14.4)	16.7(15.1)	17.0(15.3)
Self-reported quantity(pouches used/week)	5.1 (2.7)	6.3 (3.3)	5.7(3.0)
FTND-ST OSSTD	6.0 (1.9) 33.3 (8.3)	6.3 (2.1) 29.6 (8.2)	6.1 (2.0) 31.5 (8.4)

CI = confidence interval; FTND-ST = Fagerstörm Tobacco and Nicotine Dependency Scale for Smokeless Tobacco; OSSTD = Oklahoma Scale for Smokeless Tobacco Dependence; SD = standard deviation; ST = smokeless tobacco.

All participants reported using “naswar.” Twenty participants were dual users. All dual users smoked cigarettes occasionally. The mean number of pouches of naswar consumed per week at baseline was 5.7 (SD ±3.0), with a range of 1–14 pouches/week. The mean frequency of daily use was 17 times/day (SD ±15.3), with a range of 2–60 times/day. The mean age of initiation of ST use was 19.7 years (SD ±9.1), with a range of 5–60 years. Seventy-one participants had a quit attempt during their lifetime and 31% had attempted to quit naswar in the past 12 months. Overall, there was a good balance with respect to age, ST-use behavior (frequency and quantity of ST use), and nicotine dependence, which are presented by the randomization group in Table 2.

### Participant Follow-Up

Twenty-two participants were lost to follow-up. A detailed breakdown of the withdrawals and losses to follow-up is provided in Table S1. Seventy-eight (78%) participants were successfully followed up telephonically at 6 months; this included 39 from each randomization group. Of the 16 participants who reported 6-month abstinence, 14 visited the study site for saliva sample collection.

### Participant Compliance

The compliance with study visits, by hospitals and departments, is described in Tables 2–4. Compliance of the participants was determined by attendance in the second and third hospital visits. Attendance in second and third visits was 71% and 63%, respectively. While only slight differences were observed between the randomization groups, more obvious differences were observed on the basis of the department and hospital. For instance, overall good compliance was observed in prosthodontics and periodontics departments. Whereas, poor compliance was observed in the Department of Endodontics, at both study sites. Between the study sites, higher compliance was observed at SBDC as compared to KCD. The average window period between the first and second visits was 17.5 days (SD ±13.1). The average window period between the second and third visit was 18.7 days (SD ±14.0). The Russel Standards could not be adhered to, for the window periods between the visits and follow-ups, including for biochemical verification of quit rates, because the second and third visits were scheduled by the dentist, according to the patients’ treatment plan.

Compliance to the intervention was assessed on the basis of return of the self-monitoring calendars and attendance in all three sessions. Compliance with the intervention was moderate with 64% of participants attending all intervention sessions. However, 32% returned the calendars indicating a low intervention compliance with this element. Two of these calendars were returned unmarked.

### ST-Use Outcome Variables

The ST-use outcome measures for the intervention and control at baseline and the changes in the measures over the course of the study period are described in Table 3 (and Tables S5 and S6). Each outcome measure is described separately below.

**Table 3.** Summary of Smokeless Tobacco Outcome Measures

Outcome	Control		Intervention	
6-mo self-reported quit rate	8%		24%	
6-mo SC verified quit rate	4%		10%	
	Baseline (SD)	Mean change from baseline to 6 months (SD; 95% CI)	Baseline (SD)	Mean change from baseline to 6 months (SD; 95%CI)
Quantity of ST use (weekly)	5.1 (2.7)	2.6 (3.7; 1.37, 3.8)	6.3(3.3)	4.7 (4.1; 3.4, 6.1)
Frequency of ST use (daily)	15.3 (14.4)	7 (15.7; 1.9, 12.1)	18.7(16.24)	13 (14.6;8.17, 17.8)
	Baseline (SD; 95% CI)	Mean change from baseline to third visit (SD; 95% CI)	Baseline (SD; 95% CI)	Mean change from baseline to third visit (SD; 95% CI)
FTND-ST	6.0 (1.9; 5.4,6.5)	2.7 (2.7;1.7,3.7)	6.3(2.1;5.6,6.9)	4.2 (3.4; 3.0,5.5)
OSSTD	33.3(8.3; 30.9, 35.7)	8.3(9.3; 4.9,11.8)	29.6(8.2; 27.3, 32.0)	16.2(12.1;11.8,20.7)

CI = confidence interval; SD = standard deviation; ST = smokeless tobacco.

#### Self-Reported Frequency of ST Use Per Day

The average self-reported frequency of use (number of times naswar was used per day) at baseline was 17.0 times/day (SD ±15.3) with a range of 2–60 times. A decrease in this frequency was observed in both groups, with a more rapid decline observed in the intervention group from baseline to second visit, which was maintained throughout the study period. The decline in the control group was rather gradual like the intervention group, and was maintained throughout the study period.

#### Self-Reported Quantity of ST Use Per Week

The average self-reported ST use (number of pouches of naswar used in a week) was 5.7 (SD ±3.08) with a range of 1–14 pouches of naswar. A decline in this weekly quantity was observed in both groups with a slightly greater decline observed in the intervention group compared to the control group at the second visit. Both the groups maintained the decline throughout the study period. However, a slight increase in the quantity of use was observed in the control group between the second and third visits.

#### Fagerstörm Test of Nicotine Dependence ST

The mean FTND-ST score at baseline for both groups was 6.17 (SD ±2.0) indicating high nicotine dependence. A rapid drop in the FTND-ST score was observed for both groups, indicating a reduction in nicotine dependence (intervention FTND-ST dropped by 4.2 points and control by 2.7 points).

#### Oklahoma Scale of Smokeless Tobacco Dependence

The mean OSSTD score at baseline for both groups was 31.5 (SD ±8.4), indicating high nicotine dependence. As with FTND-ST, a rapid drop in the OSSTD score was observed for both groups, indicating a reduction in nicotine dependence (intervention OSSTD dropped by 16.2 points and control by 8.3 points). Among the subscales, the lowest score was observed for “weight control,” indicating weight loss as the least common reason behind ST use among users belonging to KP. The highest score was observed in the subscale “affective enhancement” indicating a dependence on naswar for affective enhancement.

#### Smokeless Tobacco Abstinence

Assessment of abstinence was done through self-reporting and biochemical verification through SC. There were 25 (25%) self-reported quitters at the third visit, which reduced to 16% at the 6-month follow-up. A saliva sample was collected from all 4 patients in the control group and from 10 out of 12 patients who self-reported 6-month abstinence. Self-reported and biochemically verified 6-month abstinence is presented in Table 4.

**Table 4.** Self-Reported Abstinence by Randomization Group

*n*/%	Intervention control	Intervention
Self-reported abstinence at third visit	6(12%)	19(38%)
Self-reported 3-mo abstinence at 3-mo follow-up	4(8%)	17 (34%)
Self-reported six abstinence at 6 mo	4(8%)	12(24%)
Biochemically verified 6-moabstinence	2(4%)	5(10%)

## Discussion

Our study assessed the feasibility of a dentist-delivered, behavioural support intervention for ST cessation. It provides several important implications for a future definitive study. The study achieved its target of recruiting 100 patients who were ST users and were visiting the selected departments for a dental treatment, requiring multiple visits. Seventy-eight participants continued to engage throughout the study duration and provided primary outcome data. Sixty-three (63%) of the participants completed all three visits. ST-use outcome measures demonstrated an overall reduction from baseline, with a biochemically verified quit rate of 10% in the intervention group and 4% in the control group. Previous research in this field largely lacks a detailed account of participant recruitment and retention^39,40^; however, the 13% eligibility rate and 88% consent rate of our study are comparable (albeit slightly higher) to previously reported rates in similar studies.^41^ Virtanen et al.,^41^ for instance, reported a consent rate of 75%, whereas Holliday et al.^42^ reported a consent rate of 67% and an eligibility rate of 7%. A possible explanation for the slightly higher consent rate could be that our trial was conducted at teaching hospitals, which charge patients at a very nominal rate and the patients’ initial contact in our trial was with dentists. The higher consent rate might be reflective of the patients’ willingness to follow the dentists’ suggestion or the patients’ feeling of having to follow their dentists’ suggestion about trial participation. Likewise, the broad eligibility criteria of our study might have contributed to our study’s higher eligibility rate. For instance, our study recruited patients requiring a range of dental treatments and we did not exclude patients on the basis of medical conditions. Whereas, Holliday et al.^42^ limited recruitment to patients requiring periodontal treatments and excluded patients who had many medical conditions. Recruiting from a range of specialty departments allowed for a pragmatic approach and also accelerated recruitment rate, which is an important point for future trials to consider.

The 22% 6-month attrition rate of our study is in keeping with the previous literature conducted in this field. A 25% 1-year attrition rate was reported by Walsh et al.^43^ and 27% 6-month attrition rate was reported by Holliday et al.^42^ In another pilot study by Siddiqui et al.,^16^ which developed and piloted the intervention tested in our study, an attrition rate of 38% was reported.

Compliance with the trial procedures (trial visits for both groups and intervention sessions for intervention group) in our study was assessed by the participants’ attendance in trial visits. Keeping with a pragmatic approach, all visits were scheduled alongside the patients’ dental appointments, which is as would be expected in usual care. This meant that participants who missed their dental appointments also resulted in them missing their trial visits (and intervention sessions). Therefore, the participants’ compliance with the visits (and intervention sessions) cannot be solely viewed as their compliance with the trial procedures, rather it also reflects their compliance with their dental treatment. All participants who showed up for their dental appointments also completed their scheduled trial visit and intervention sessions. With regards to participants’ compliance across the different departments, overall good compliance was observed in the prosthodontics and periodontics and poor compliance in the department of endodontics, at both the study sites. As dental treatments require multiple visits, therefore, it is unlikely that the burden of visits contributed to the participant attrition rate. One possible explanation behind low retention in the endodontics department, could be that this department, was the busiest department in terms of patient load, with patients often having to wait for weeks and even months (at KCD) to get their treatment started. Due to these long waiting times, patients often times seek treatment from other hospitals/clinics. Future trials using the same intervention should consider whether the number of visits can be reduced to two, in which case, the return of the calendars can be considered via mobile phones or other mediums. Another approach for overcoming the issue of noncompliance is incentivizing trial participation and facilitating the dental treatment of the participants. For instance, by ensuring that they get timely appointments with less waiting time, etc., for which a liaison with the hospital administration may be considered.

The recent Cochrane systematic review on interventions for tobacco cessation in dental settings included seven studies on multisession behavioral support for tobacco cessation by dental professionals. Only one of these reported biochemically verified quit rates. Findings from these studies, subgrouped by motivation, reported 11.9% 6-plus-month abstinence rate (self-reported), from six studies in which participants were not selected for motivation. Whereas, one study which recruited participants who were motivated to quit reported a 6-month abstinence rate of 14% (laboratory verified).^17^ This review also included three studies involving multisession behavioral intervention for “ST” cessation.^39,44,45^ The self-reported 6-plus-month abstinence from these studies was 11.4%. One study involving both smoked and ST users reported a laboratory verified 6-month abstinence of 14%.^17^ However, as mentioned, this study only recruited participants who intended to quit. Literature suggests a reduction in quitters with increasing strictness of tobacco abstinence measures.^46^ The same was noted in the current study as the abstinence rate dropped by 50% with biochemical verification. Nonetheless, the intervention group in the current study achieved a comparable abstinence rate of 10%. While recruitment on the basis of intention to quit might have resulted in higher quit rates in our study, such eligibility criteria would not have been in line with the universal approach of tobacco cessation. The saliva samples were collected without problems from several participants; however, there was considerable delay in the collection of samples from others who had moved out of town. Future trial designs could consider mailing the salivette to the participation for collection of samples at home, rather than having the patients visit the study sites for collection.

The generalizability of the findings from this pilot study is limited because the trial was conducted at two dental hospitals. The selected hospitals were the largest tertiary-level teaching hospitals, catering to all kinds of patients and the level of work ranged from the simplest of tasks like taking a dental radiograph to the most expansive maxillofacial surgeries, hence these catered to a large and diverse catchment, reflective of the general population. Within these hospitals, the trial was conducted at three different specialty departments, which further allowed for diversity in dental patients, dental treatments, and the ward setting at each department. Nonetheless, the setting of this study may not be representative of all public dental settings.

Another factor limiting the generalizability of the findings is the lack of female representation in the trial sample, as only one female was recruited who was lost to follow-up. With 3.7% of females using ST in Pakistan, the female representation in the sample should have been close to 20%. The lack of female representation in research on ST from this region is, however, not an uncommon finding, as previous studies from KP involving ST users, were not successful in recruiting female ST users.^47,48^ This issue can be possibly attributed to sociocultural influences. To this end, a qualitative study was conducted in preparation for this pilot trial, to explore the barriers and facilitators for ST cessation support in dental settings in Pakistan.^49^ The dentists interviewed in the study and reported never asking a female patient about ST use, due to sociocultural norms and inquiring about ST from female patients was reportedly accompanied by emotions of fear and embarrassment. Perhaps the hesitance among the dentists to inquire about ST status from female patients led to an underrepresentation of females in the trial sample, as the identification of potential participants in the trial was done by the dentists. The qualitative study also involved interviews with dental patients (who were ST users) and the representation of females in the sample was 25%. All females acknowledge the need for ST cessation support in dental settings. These findings suggest the relevance of the intervention for female ST users; however, the issue might lie in the identification of female users. Another possible reason for the underrepresentation of females in the sample, could be the limited access of females to dental health care. The relevance of the intervention for females, therefore, remains undecided, which highlights the need for further research for a deeper understanding of the issue. To address the limitations in generalizability of the trial findings, future trials should consider primary and secondary care settings and consider other sampling techniques (such as snowballing) for the recruitment of female ST users to ensure a wider applicability of the results.

Overall, the trial demonstrated that it is feasible to offer a dentist-delivered behavioral support intervention during routine clinical practice. The study offers several design implications for a future definitive study. These include expected eligibility, recruitment, and retention rates; incentivizing trial participation, facilitating participants in their dental treatment, not including the patients’ intention to quit as an inclusion criterion; study design to be highly pragmatic (broad inclusion criteria, conducted in all specialty departments and all levels) and reducing the number of visits.

## Conclusions

It was feasible to deliver a structured behavior support intervention for ST cessation to dental patients via dentists in dental settings in Pakistan. The data suggested that the intervention may improve ST quit rates. The findings of this study can inform the design of a future definitive study.

## Supplementary Material

Supplementary material is available at *Nicotine and Tobacco Research* online.

ntad243_suppl_Supplementary_Appendixs_1

ntad243_suppl_Supplementary_Appendixs_2

ntad243_suppl_Supplementary_Appendixs_3

ntad243_suppl_Supplementary_Appendixs_4

ntad243_suppl_Supplementary_Appendixs_5

ntad243_suppl_Supplementary_Material

## Data Availability

The data generated and analyzed during the study are available from the corresponding author upon reasonable request.
