# Supplementary material for: Behavior Change Intervention for Smokeless Tobacco Cessation Delivered Through Dentists in Dental Settings: A Pragmatic Pilot Trial
Source: Nicotine Tob Res. 2023 Dec 11;26(7):878–87. doi: 10.1093/ntr/ntad243 (PMC11190057; doi:10.1093/ntr/ntad243)
Supplement: ntad243_suppl_Supplementary_Appendixs_1 [file ntad243_suppl_supplementary_appendixs_1.docx]

Appendix 1. Eligibility criteria

The trial participants were dental patients who were seeking dental care at the selected study sites and selected departments.

The follow eligibility criteria was used to recruit the trial participants:

Inclusion criteria

1. 18 years and above.
2. Regular smokeless tobacco users (regular use = at least once in 7 days for 6 months or more)(1).
3. Willingness to visit the study site multiple times.
4. Willing and able to provide written informed consent.

Exclusion criteria

1. Currently accessing cessation support.
2. Less than 18 years
3. Unwilling or unable to provide written informed consent.

Dentists were recruited in this study for intervention delivery. The following eligibility criteria was used to recruit the dentists:

Inclusion Criteria

1. Having completed four year ‘Bachelors of Dental Surgery’ (BDS) degree and one-year house job training.

2. Working in the periodontics or prosthodontics departments of KCD or SBDC.

3. Willing and able to provide written informed consent.

Exclusion Criteria

1. Working in departments other than prosthodontics and periodontics departments of KCD or SBDC.
2. Those who have not completed one-year house job training.
3. Unwilling or unable to provide written informed consent.

1. Siddiqi K, Dogar O, Rashid R, Jackson C, Kellar I, O’Neill N, et al. Behaviour change intervention for smokeless tobacco cessation: its development, feasibility and fidelity testing in Pakistan and in the UK. BMC public health. 2016;16(1):1-15.
