# Supplementary material for: Behavior Change Intervention for Smokeless Tobacco Cessation Delivered Through Dentists in Dental Settings: A Pragmatic Pilot Trial
Source: Nicotine Tob Res. 2023 Dec 11;26(7):878–87. doi: 10.1093/ntr/ntad243 (PMC11190057; doi:10.1093/ntr/ntad243)
Supplement: ntad243_suppl_Supplementary_Appendixs_3 [file ntad243_suppl_supplementary_appendixs_3.docx]

Appendix 3. Protocol approval and amendments

| **Protocol Version** | **Amendment** | **Details** | **REC Reference**  **Approved date** |
| --- | --- | --- | --- |
| v1.1 | Original submission |  | 21-EMREC-024  6.9.2021 |
| v2.0 | Protocol changes | Non-substantial changes were made to the protocol following REC approval. These included changes in the intervention resources and changes in recruitment plan. To allow for recruitment of participants from endodontics and maxillofacial departments in addition to the previously agreed department if the recruitment is slow. In addition to this to allow for a 70:30 or 60:40 ratio of participants recruitment between KCD and SBDC as opposed to previously agreed 50:50 ratio. | 21-EMREC-024- NSA01  10.1.2022 |
| v3.0 | Protocol changes | Substantial changes were made which included addition of collection of saliva samples for cotinine analysis at 6 months follow up. | 21-EMREC-024_SA01  15.6.2022 |
