# Supplementary material for: Behavior Change Intervention for Smokeless Tobacco Cessation Delivered Through Dentists in Dental Settings: A Pragmatic Pilot Trial
Source: Nicotine Tob Res. 2023 Dec 11;26(7):878–87. doi: 10.1093/ntr/ntad243 (PMC11190057; doi:10.1093/ntr/ntad243)
Supplement: ntad243_suppl_Supplementary_Appendixs_4 [file ntad243_suppl_supplementary_appendixs_4.docx]

Appendix 4. TIDieR Checklist- Intervention

| **S. No.** | **Item** | **Definition** |
| --- | --- | --- |
| 1. | Brief name | Behavioural support Intervention for Smokeless Tobacco Cessation in South Asians(BISCA) (33) |
| 2. | Why? | Focused face-to-face behavioural support for tobacco cessation is very effective in increasing quit rates (Relative Risk (RR): 1.57, 95%CI: 1.40 to 1.77) (51).  Dentists are the first to notice any changes in the oral mucosa due to tobacco use. Most of the treatments require more than one visit. These factors place dentists in a unique opportunist position to identity tobacco users and offer quit advice and support for tobacco cessation.  It is recommended that provision of quit advice and support by dentists should be integrated into their routine clinical practice. |
| 3. | What  (materials)? | *Participants (users):* Self-help calendars and take home booklet for the patients. The purpose of the booklet was to reinforce the messages delivered in the sessions. Whereas, the calendar was given for self-monitoring purpose ‘offered at the quit-session’.  *Intervention providers (dentists*): Practice manual and flipbooks were given to the participating dentists. |
| 4. | What  (procedures)? | Structured behaviour support ‘face-to-face counselling’ was delivered by dentists in three sessions namely pre-quit, quit and post-quit sessions.  *Pre-quit session.* The activities included in the pre-quit session aimed to develop the users’ intention to change and to plan and prepare the user for quitting. *Quit session.* The activities included in this session were centred on a reflection on the preparation and support on the quit day. *Post quit session:* The purpose of this session was provision of ongoing support and confirmation of abstinence. |
| 5. | Who will provide? | Dentists trained in delivering BISCA. |
| 6. | How? | BISCA was delivered as an individual face-to-face conversation. |
| 7. | Where? | Periodontics, prosthodontics and endodontics departments of KCD and SBDC. |
| 8. | When and  how much? | BISCA was delivered in three sessions. Each session was delivered before or after the dental treatment was completed (as deemed appropriate by the dentist). The mean duration of the pre-quit sessions was 19.6 minutes with a range of 6.4 to 29.6 minutes, the mean duration of the quit sessions was 6.9 minutes with a range of 1.5 to 13.1 minutes and the mean duration of the post-quit session was 2.7 minutes with a range of 0.6 to 4.3 minutes. On average the ‘quit’ session were scheduled 17 days after the pre-quit session whereas the post-quit session was scheduled 18 days following the ‘quit’ session. |
| 9 | Tailoring | The conversation was tailored to the specific type of ST product used by the patient. Furthermore, the quit advice in the pre-quit-session and continued support offered in the subsequent sessions was guided by the level of patients’ motivation and engagement. The duration of the sessions was among other things dependent upon the interaction between the dentists and patient |
| 10 | Modification | N/A |
| 11. | How well  (planned) | One-day workshop (at each site) was arranged to train all participating dentists to deliver BISCA. Training manual was given to the dentists and they were strongly advised to adhere to the manual for delivering the intervention. Fidelity to intervention delivery was assessed via audiotaping the interactions between the dentists and patients. |
| 12. | How well  (actual) | N/A |
