# Supplementary material for: Behavior Change Intervention for Smokeless Tobacco Cessation Delivered Through Dentists in Dental Settings: A Pragmatic Pilot Trial
Source: Nicotine Tob Res. 2023 Dec 11;26(7):878–87. doi: 10.1093/ntr/ntad243 (PMC11190057; doi:10.1093/ntr/ntad243)
Supplement: ntad243_suppl_Supplementary_Appendixs_5 [file ntad243_suppl_supplementary_appendixs_5.docx]

Appendix 5.TIDieR Checklist- Self-help material (control)

| **No** | **Item** | **Definition** |
| --- | --- | --- |
| 1. | Brief name | Self-help material |
| 2. | Why? | Self-material has some benefit of increasing quit rates in comparison to no intervention (12 trials, RR 1.21; 95% CI 1.05 to 1.39) (52). |
| 3. | What (materials)? | Take home booklet (the same booklet given to intervention group) |
| 4. | What (procedures)? | All patients were offered the booklet in the first visit. |
| 5. | Who provided? | Dentists |
| 6. | How? | The booklet was handed over to the participants by the dentist during the first visit. |
| 7. | Where? | Periodontics, prosthodontics and endodontics departments of KCD and SBDC |
| 8. | When and  how much? | First visit(one booklet) |
| 9 | Tailoring | N/A |
| 10 | Modification | N/A |
| 11. | How well (planned) | N/A |
| 12. | How well(actual) | N/A |
