# Supplementary material for: Behavior Change Intervention for Smokeless Tobacco Cessation Delivered Through Dentists in Dental Settings: A Pragmatic Pilot Trial
Source: Nicotine Tob Res. 2023 Dec 11;26(7):878–87. doi: 10.1093/ntr/ntad243 (PMC11190057; doi:10.1093/ntr/ntad243)
Supplement: ntad243_suppl_Supplementary_Material [file ntad243_suppl_supplementary_material.docx]

Supplementary table 1. Details of withdrawals and loss to follow up

| **Participant ID** | **Study Arm** | **Study visits and follow ups completed** | **Reason** |
| --- | --- | --- | --- |
| PID8 | Control | 1^st^ , 2^nd^ and 3^rd^ visit and three month follow-up | Cannot be reached |
| PID13 | Intervention | 1^st^ visit and three month follow up | Cannot be reached |
| PID12, PID80, PID100 | Intervention | 1^st^ visit | Cannot be reached |
| PID26 | Intervention | 1^st^,2^nd^ and 3^rd^ visit | Cannot be reached |
| PID20, PID30, PID98 | Control | 1^st^ | Cannot be reached |
| PID 64 | Control | 1^st^, 2^nd^ and 3^rd^ visits and three month follow up | Cannot be reached |
| PID 68 | Intervention | 1^st^ and 2^nd^ visit | Cannot be reached |
| PID73 | Intervention | 1^st^,2^nd^, 3 and 3 month follow up | Left country |
| PID77, PID96 | Control | 1^st^ visit and three month follow up | Cannot be reached |
| PID43,PID59, PID65 PID84 | Control | 1^st^ and 2^nd^ visit and three month follow-up | Cannot be reached |
| PI54,PID70, PID88,PID93 | Intervention | 1^st^, 2^nd^ and 3^rd^ visit and three month follow up | Cannot be reached |

Supplementary table 2. Compliance with study visit-2 and duration between baseline and 2^nd^ visit by hospital and department

| **n=attendance**  **Mean**  **SD**  **Min**  **Max**  **95%CI** | **KCD** | **SBDC** | **Total** |
| --- | --- | --- | --- |
| Prosthodontics | n=23  Mean=19.9  SD±14.5  Min=4  Max=58  95%CI=13.6, 26.2 | n=9  Mean=18  SD±18.2  Min=5  Max= 62  95%CI= 4.0, 31.9 | n=32  Mean=19.4  SD±15.3  Min= 4  Max=62  95%CI= 13.8, 24.9 |
| Periodontology | n=11  Mean= 16.5  SD±9.2  Min=6  Max=34  95%CI=10.3, 22.7 | n=5  Mean=9  SD±2.7  Min= 5  Max=12  95%CI=5.5, 12.4 | n=16  Mean= 14.1  SD±8.4  Min= 5  Max=34  95%CI=9.6, 18.7 |
| Endodontics | n=9  Mean=19.1  SD±13.3  Min=7  Max= 49  95%CI=8.8, 29.3 | n= 14  Mean=16.2  SD±2.11  Min= 5  Max=51  95%CI=9.2, 23.2 | n=23  Mean=17.3  SD±12.3  Min=5  Max=51  95%CI=11.9, 22.7 |
| Total | n=43  Mean=18.9  SD±12.9  Min=4  Max=58  95%CI= 14.9, 22.8 | n=28  Mean=15.5  SD±13.4  Min= 5  Max=62  95%CI= 0.2, 20.7 | n=71  Mean=17.5  SD±13.1  Min=4  Max=62  95%CI=14.4, 20.6 |

Supplementary table 3. Compliance with study visit-3 and duration between 2^nd^ and 3^rd^ visit by hospital and department

| **Mean**  **SD**  **Min**  **Max**  **95%CI** | **KCD** | **SBDC** | **Total** |
| --- | --- | --- | --- |
| Prosthodontics | n=20  Mean=16.7  SD±14.9  Min= 2  Max=57  95%CI=9.7, 23.7 | n=9  Mean=19.6  SD±11.4  Min=8  Max=40  95%CI=10.9, 28.4 | n= 29  Mean=17.6  SD±13.8  Min= 2  Max=57  95%CI=12.3, 22.9 |
| Periodontology | n= 9  Mean=15.6  SD±9.0  Min= 6  Max= 35  95%CI= 8.6, 22.5 | n=5  Mean=24.2  SD±22.8  Min= 6  Max= 63  95%CI=-4.0, 52.5 | n= 14  Mean=18.6  SD±15.1  Min= 6  Max=63  95%CI=9.9 , 27.3 |
| Endodontics | n=8  Mean=18  SD±13.1  Min=6  Max=43  95%CI=7.0, 28.9 | n=12  Mean=21.8  SD±15.40  Min=3  Max= 47  95%CI=12.0, 31.6 | n=20  Mean=20.3  SD±14.3  Min=3  Max=47  95%CI=13.60, 26.9 |
| Total | n=37  Mean=16.7  SD±13.0  Min=2  Max=57 | n=26  Mean=21.5  SD±15.2  Min=3  Max= 63  95%CI=15.3, 27.6 | n=63  Mean=18.7  SD±14.0  Min=2  Max= 63  95%CI=15.1, 22.2 |

Supplementary table 4. Attendance and duration between visits by randomisation group

| **Mean**  **SD**  **Min**  **Max**  **95%CI** | **Intervention(n)** | **Control(n)** | **Total(n)** |
| --- | --- | --- | --- |
| Visit 1(Baseline) | 50 | 50 | 100 |
| Attended 2^nd^ visit | 36 | 35 | 71 |
| Did not attend 2^nd^ visit | 14 | 15 | 29 |
| Time (no. of days) between baseline and 2^nd^ visit | Mean= 19.7  SD=±16.0  Min=4  Max=62  95%CI=14.2, 25.1 | Mean=15.3  SD=±8.8  Min=5  Max=49  95%CI=12.2, 18.3 | Mean=17.5  SD=±13.1  Min=4  Max=62  95%CI=14.4, 20.6 |
| Attended 3^rd^ visit | 32 | 31 | 63 |
| Participants who attended 2^nd^ visit but did not attend 3^rd^ visit. | 4 | 4 | 8 |
| Time(days) between 2^nd^ and 3^rd^ visit | Mean=16.4  SD±14.0  Min=2  Max=63  95%CI=11.3, 21.4 | Mean=21.0  SD±13.9  Min=3  Max=47  95%CI=15.99, 26.1 | Mean=18.7  SD±14.0  Min=2  Max=63  95%CI=15.1, 22.2 |

Supplementary table 5. Smokeless tobacco use outcome measures (Control group)

| **Outcome**  **Mean**  **Min**  **Median**  **Mean(SD)**  **Max**  **95%CI** | **Baseline(all)** | **Baseline(for those attending 2^nd^ visit)** | **Baseline for those attending 3^rd^ visit** | **Visit2** | **Visit3** | **3 month follow-up** | **6 month follow up** |
| --- | --- | --- | --- | --- | --- | --- | --- |
| Self-reported daily frequency of use | n=50  Mean=15.3 SD±14.4  Min=3  Max=60  95%CI= 11.2, 19.4 | n=35  Mean=14.8 SD±114.8  Min=3  Max= 60  95%CI= 9.4,19.9 | n=31  Mean=15.0  SD±15.3  Min=3  Max= 60  95%CI=9.4, 20.6 | n=35  Mean=9.571 SD±12.2841 Min=0  Max=50  95%CI=5.35, 13.7 | n=31  Mean=9.2 SD±12.6 Min=0 Max=50  95%CI=4.6, 13.9 | n=41  Mean=9.4  SD±9.7  Min=0  Max=40  95%CI= 6.4, 12.5 | n=39  Mean=8.9  SD=10.4  Min=0  Max-40  95%CI=5.5, 12.3 |
| Self-reported Weekly quantity | n=49  Mean=5.1  SD±2.7  Min=1  Max= 14  95%CI= 4.3, 5.8 | n=34  Mean=5.0  SD±2.8  Min=1  Max= 14  95%CI= 4.1, 6.0 | n=30  Mean=5.1  SD±2.8  Min=1  Max= 14  95%CI=4.0, 6.2 | n=35  Mean=2.8 SD±2.6  Min=0  Max=7  95%CI=1.9, 3.7 | n=31  Mean=3.1 SD±3.0 Min=0 Max=14  95%CI=2.0, 4.2 | n=41  Mean=3.4  SD±3.2  Min=0  Max=14  95%CI=2.4, 4.4 | n=39  Mean=2.8  SD=3.3  Min=0  Max=14  95%CI=1.7, 3.9 |
| FTND-ST | n=49  Mean=6.0 SD±1.9  Min= 0  Max=9  95%CI= 5.4, 6.5 | n=34  Mean= 5.9  SD±1.9  Min=0  Max=9  95%CI= 5.2, 6.6 | n=30  Mean=6  SD±2.0  Min= 0  Max=9  95%CI= 5.2, 6.7 | - | n=31  Mean=3.0  SD±2.8 Min=0 Max=8  95%CI= 2.0, 4.1 | - |  |
| OSST | n=50  Mean=33.3 SD±8.3  Min=7.5  Max=42.6  95%CI | n=35  Mean=33.1  SD±9.4  Min=7.5  Max=42.6  95%CI=29, 36.4 | n=31  Mean=32.5  SD±9.7  Min=7.5  Max=42.6  95%CI=28.9, 36.1 | - | n=31  Mean=24.1 SD±11.5 Min=7  Max40.3  95%CI=19.9, 28.4 | - |  |

Supplementary table 6. Smokeless tobacco use outcome measures (Intervention group)

| **Outcome**  **Mean**  **Min**  **Median**  **Mean(SD)**  **Max**  **95%CI** | **Baseline (all)** | **Baseline (for those attending 2^nd^ visit)** | **Baseline for those attending 3^rd^ visit** | **Visit2** | **Visit3** | **3 month follow-up** | **6 month follow-up** |
| --- | --- | --- | --- | --- | --- | --- | --- |
| Self-reported daily frequency of use | n=49  Mean=18.7  SD±16.24  Min=2  Max=60  95%CI=14.0,19.8 | n=35  Mean=17  SD±15.2  Min=3  Max=60  95%CI=12.6, 23.1 | n=31  Mean=17.8  SD±14.7  Min=3  Max=60  95%CI=12.4, 23.2 | n=36  Mean=5.4  SD±11.4 Min=0  Max=65  95%CI=1.5, 9.3 | n=32  Mean=3.8 SD±6.7 Min=0 Max=25  95%CI=1.4, 6.2 | n=41  Mean=5.2  SD±10.4  Min=0  Max=50  95%CI=1.9, 8.5 | n=39  Mean=4.3  SD=6.9  Min=0  Max=25  95%CI=2.0, 6.5 |
| Self-reported Weekly quantity | n=49  Mean=6.3  SD±3.3  Min=1.5  Max=14  95%CI=5.3,7.2 | n=35  Mean=6.6  SD±3.30  Min=1.5  Max=14  95%CI=5.4, 7.7 | n=31  Mean=6.6  SD±3.0  Min=2  Max=14  95%CI=5.4, 7.7 | n=36  Mean=2.1 SD±3.0, Min=0  Max=14  95%CI=1.1, 3.21 | n=32  Mean=1.5 SD±3.0 Min=0 Max=14  95%CI=0.4, 2.6 | n=41  Mean=1.8  SD±2.5  Min=0  Max=7  95%CI=1.0, 2.6 | n=39  Mean=1.4  SD=2.1  Min=0  Max=7  95%CI=0.7, 2.1 |
| FTND-ST | n=50  Mean= 6.3 SD±2.1  Min=0  Max=10  95%CI=5.6, 6.9 | n=36  Mean=6.6  SD±1.9  Min=1  Max=10  95%CI=6.0, 7.3 | n=32  Mean= 6.5  SD±2.0  Min=1  Max= 10  95%CI=5.8, 7.3 | - | n=31  Mean=2.1 SD±3.0 Min=0 Max=8  95%CI=1.0, 3.2 | - |  |
| OSSTD | n=50  Mean=29.6 SD±8.2  Min=8  Max=41.0  95%CI=27.3, 32.0 | n=36  Mean=31.3 SD±7.5  Min=11.1 Max=41.0  95%CI=28.7, 33.8 | n=32  Mean=32.4  SD±6.5  Min=17.7  Max=41.0  95%CI=30.1, 34.8 | - | n=31  Mean=16.1 SD±11.2 Min=7  Max= 43  95%CI=12.0, 20.3 | - |  |
